# Instability of standard PCR reference genes in adipose-derived stem cells during propagation, differentiation and hypoxic exposure

**DOI:** 10.1186/1471-2199-9-98

**Published:** 2008-10-31

**Authors:** Trine Fink, Pia Lund, Linda Pilgaard, Jeppe Grøndahl Rasmussen, Meg Duroux, Vladimir Zachar

**Affiliations:** 1 Laboratory for Stem Cell Research, Aalborg University, Denmark; 2 Department of Pharmacology, University of Aarhus, 8000 Aarhus C, Denmark

## Abstract

**Background:**

For the accurate determination of gene expression changes during growth and differentiation studies on adipose-derived stem cells (ASCs), quantitative real-time RT-PCR has become a method of choice. The technology is very sensitive, however, without a proper selection of reference genes, to which the genes of interest are normalized, erroneous results may be obtained.

**Results:**

In this study, we have compared the gene expression levels of a panel of twelve widely used reference genes during hypoxic culture, osteogenic and chondrogenic differentiation, and passaging of primary human ASCs. We found that several of the commonly used reference genes including 18S rRNA, glyceraldehyde-3-phosphate dehydrogenase (GAPDH) and beta-actin were unsuitable for normalization in the conditions we tested, whereas tyrosine 3/tryptophan 5-monooxygenase activation protein (YMHAZ), TATAA-box binding protein (TBP), beta-glucuronidase (GUSB) were the most stable across all conditions.

**Conclusion:**

When determining gene expression levels in adipose-derived stem cells, we recommend normalizing transcription levels to the geometric mean of YMHAZ, TBP and GUSB.

## Background

In the research on mesenchymal stem cells adipose tissue-derived stem cells (ASCs) are increasingly being used as an alternative cell source to bone marrow-derived stem cells [1]. As the bone marrow-derived cells the ASCs have the potential to differentiate into osteocytes and chondrocytes, as well as other multiple other cell types [2].

For the monitoring of gene expression during routine culture of primary ASCs and in studies on their differentiation, quantitative PCR is often the method of choice, due to its reliability, affordability and ease to perform. However, the validity of the gene expressions determined by this method is heavily dependant on the optimal selection of reference genes to which the genes of interest are to be normalized. The importance of selecting appropriate reference genes is often overlooked. However, normalizing to genes that are responsive to the test conditions will lead to erroneous results [3]. The expression of an optimal reference gene will ideally be unchanged in the cells irrespective of the experimental conditions and the level of expression should also be similar to the expression level of the genes of interest. To further minimize the risk of bias due to variations of expression levels in a single housekeeping gene, several groups have suggested that at least two housekeeping genes should used for normalization purposes [4,5].

Traditionally, reference genes include structural genes such as beta-actin or genes involved in metabolism such as glyceraldehyde 3-phosphate dehydrogenase (GAPDH). However, during cell differentiation, genes that are involved in the modulation of the cytoskeleton or metabolic pathways are often changed, rendering these genes useless for normalization purposes [6,7]. In addition, ribosomal RNA is often used for normalization, as the total amount of ribosomal RNA is believed to be relatively stable. When determining the levels of 18S rRNA, however, some groups compare the amounts of 18S rRNA between samples where the input load of total RNA has been adjusted. As the ribosomal RNA makes up for the bulk of RNA in the samples, such an approach will invariably lead to the assumption that the rRNA levels in the cells are unchanged. In fact, several studies have shown that the levels of ribosomal RNA do vary under some conditions [8-10]. Yet another problem in normalizing to ribosomal RNA content, is that the very high levels of ribosomal RNA make it difficult to establish a baseline value in most real-time PCR protocols.

Recently, studies showed that several genes normally used as reference genes had varying expression levels in differentiating embryonic stem cells [11,12]. Also, two studies on adipose-derived cells lead to contrasting results. In one study on cultured adipocytes and preadipocytes exposed to different hormones, the authors found GAPDH and transferrin receptor to be the least changed [13], whereas another study on adipose tissue biopsies found 18S rRNA to be the most and GAPDH to be the least suitable reference genes [14]. While it seems unlikely that we can identify a gene whose expression is constant under all phases through the cell cycle and under any differentiation conditions, it is feasible to select reference genes that are stable under a subset of selected conditions. Thus, the purpose of this study was to select a set of reference genes, which are suitable as internal standards in studies on adipose-derived mesenchymal stem cell growth and differentiation. We have studied the expression of 12 often used reference genes during the first five passages of primary ASCs, after osteogenic and chondrogenic differentiation of the ASCs, and after hypoxic exposure for up to two weeks.

## Results

### ASC characteristics, RNA yield and quality

**Figure 1:**
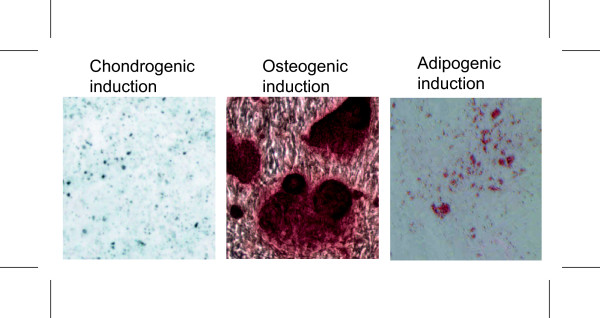
**Histochemical staining of adipose-derived stem cells subjected to chondrogenic, osteogenic, or adipogenic induction.** The chondrogenic incduction was confirmed by Alcian blue staining of the chondrocyte-specific glucosaminoglycans. The osteogenic phenotype was revealed by staining with Alizarin red, which stains calcium-rich mineral deposits. For the adipogenic cells, intracellular accumulations of lipid droplets were stained with Oil red O.

Excess human adipose tissue was obtained from two female patients, undergoing elective surgery at Grymer Privat Hospital, Skejby, Denmark in accordance with protocols approved by the regional Committee on Biomedical Research Ethics in Northern Jutland. The two donors were 56 and 44 years of age and had BMIs of 28 and 27 respectively. The ASCs were isolated from abdominal adipose tissue, and were induced to become osteocytes, chondrocytes, and adipocytes. The histochemical stainings (Fig. 1) and the gene expression studies demonstrated that the cells could differentiate towards chondrogenic, osteogenic, and adiopogenic lineages, thus confirming their identity as mesenchymal stem cells.

In addition to the differentiation procedures, the cells were grown for up to five passages, with RNA being harvested from each passage. Also, cells were exposed to hypoxia (1% oxygen) for up to two weeks.

The yield of RNA varied between the samples from 2.5 μg to 13 μg, reflecting larger cell numbers as the cells were cultured for longer periods. By spectrophotometric analysis, we ascertained that the purity of the samples were very high with A_260_/A_280 _ratios ranging from 1.93 to 2.06 (mean 2.03). The integrity of the RNA was determined by electrophoretic analysis. Using an algorithm of the Agilent software, the samples were assigned RNA integrity numbers (RIN) which describes the integrity of the RNA on a scale from 1 to 10 [15]. The integrity of the RNA was high in all samples, with RINs above 9.7 for all samples (mean 9.96). The RNA samples were thus suitable for the sensitive analysis by real-time RT-PCR.

### Ranking of the housekeeping genes and determination of optimal number for normalization

**Figure 2:**
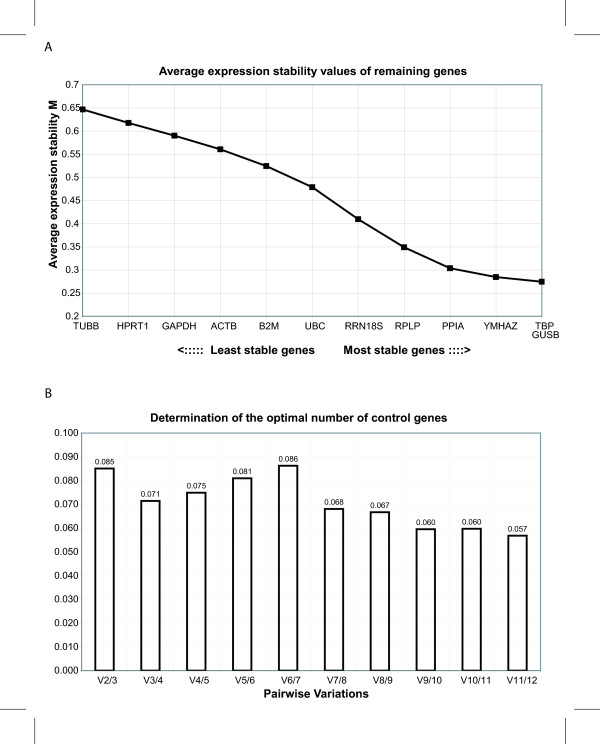
**Determination of least variable reference genes and optimal number of genes for normalization.** (A): The average expression stability measure (M) of reference genes during stepwise exclusion of least stable reference gene is shown. The genes are ranked from left to right according to increasing stability. (B): The pairwise variation V between two sequential normalization factors containing an increasing number of genes.

For the selection of the reference genes, we analyzed the genes tubulin, beta-polypeptide (TUBB), glyceraldehyde-3-phosphate dehydrogenase (GAPDH), beta-actin (ACTB), beta-2-microtubulin (B2M), ubiquitin C (UBC), 60S acidic ribosomal protein P0 (RPLP), cyclophilin A (PPIA), tyrosine 3/tryptophan 5-monooxygenase activation protein (YMHAZ), TATAA-box binding protein (TBP), beta-glucuronidase (GUSB), hypoxanthine-guanine phosphoribosyltransferase (HPRT1) as well as rRNA 18S (RRN18S), all included in the Human Endogenous Control Panel from the TATAA Biocenter (DNA-technology, Aarhus, Denmark). In order to identify the most stable reference genes across all the tested conditions, the expression levels of all reference genes were analyzed using the geNorm software. The analysis showed that the expression levels of TBP, GUSB, YMHAZ, and the PPIA varied the least across all the tested conditions, and TUBB, HPRT1, GAPDH, and ACTB varied the most (Figure 2A). Secondly, the optimal number of reference genes was assessed calculating the pairwise variation between a given number of reference genes and the inclusion of an additional gene (Fig. 2B). A large variation indicates the added gene has a significant effect and should be included. A cut-off value of 0.15 has been proposed, where the inclusion of an extra gene has little effect on the normalization [4]. As seen in figure 2B, the inclusion of three genes as opposed to two (V2/3 is 0.085) has a small effect. In the interest of an optimal determination of the variation of the other genes, we opted for the use of three reference genes (TBP, GUSB, and YMHAZ) in the further analysis.

### Variation of reference genes across different growth conditions

**Figure 3:**
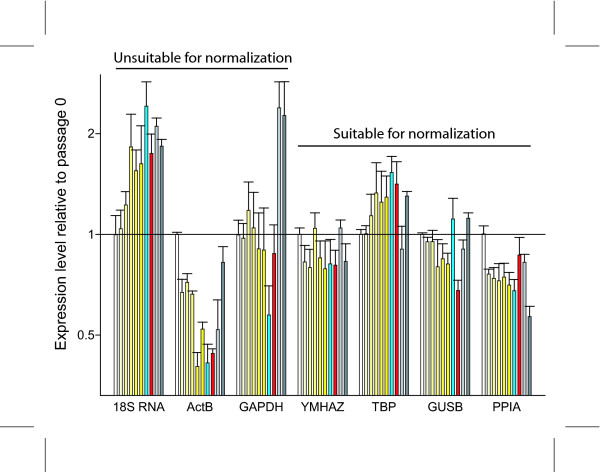
**The expression levels of putative reference genes normalized to the geometric mean of YMHAZ, TBP, and GUSB.** The values for each gene are relative to the expression level in cells at passage 0 (white bars). The yellow bars indicate subsequent passages, where the increasing intensity corresponds to the duration of culture. The blue bars denote values from chondrogenic cultures and the red indicate those from osteogenic cultures. The two light and dark grey bars represent values from cells cultured for one and two weeks in hypoxia, respectively. Abbreviations: ActB, beta-actin; GAPDH, glyceraldehyde-3-phosphate dehydrogenase; YMHAZ, tyrosine 3/tryptophan 5-monooxygenase activation protein; TBP, TATAA-box binding protein; GUSB, beta-glucuronidase; PPIA, cyclophilin A.

In order to demonstrate the differences between the highly and little variable reference genes in our study, we normalized three of the most commonly used reference genes namely 18S RNA, beta-actin and GAPDH as well as the four least variable genes to the geometric mean of TBP, GUSB, and YMHAZ (Geom mean). As shown in figure 3, the levels of 18S RNA increase up to 2-fold with increasing passages and during differentiation. Contrary to this, the levels of beta-actin decrease more than 2-fold during passaging. The levels mRNA of GAPDH, which has previously been identify as a suitable reference gene for normalization in the study of preadipocytes [13], appear to be relatively stable during passaging, however, there is a decrease of transcript during chondrogenesis and a 2-fold induction during hypoxic culture. This hypoxic upregulation is not surprising, as GAPDH has previously been shown to be highly regulated by the oxygen tension in numerous other cell types [16-18].

### Effect of choosing suboptimal reference genes

**Figure 4:**
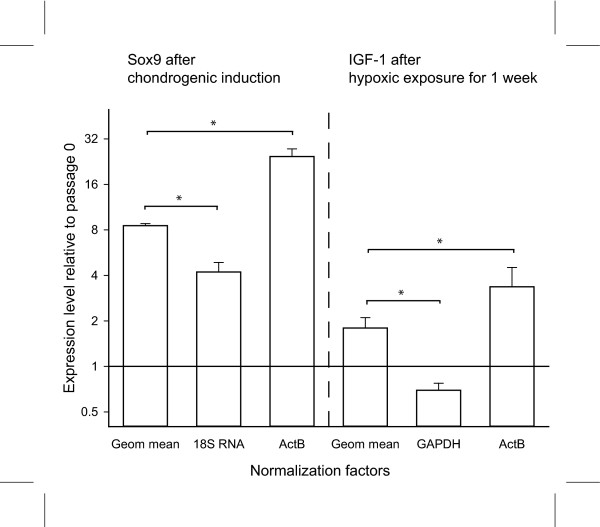
**The effect of different normalization factors on the relative gene expression levels of Sox9 and insulin-like growth factor-1 (IGF-1).** The expression level of Sox9 is presented after chondrogenic induction relative to values at passage 0. The values have been normalized to either the geometric mean (Geom mean) of tyrosine 3/tryptophan 5-monooxygenase activation protein (YMHAZ), TATAA-box binding protein (TBP), and beta-glucuronidase (GUSB), to 18S RNA, or to beta-actin (ActB). The effect of hypoxic culture on expression of IGF-1 is presented after the values have been normalized to either the geometric mean, GAPDH, or ActB levels. Error bars denote standard error of the mean, and asterisks indicate *p *< .05 compared with values normalized to the geometric mean.

To illustrate the effect of normalizing to reference genes, whose levels are not constant under the experimental condition, we have chosen to determine the expression levels of the chondrocyte-specific transcription factor Sox9 and the hypoxia-inducible growth factor IGF-1, after chondrogenic induction or hypoxic treatment, respectively. The levels of expression are normalized to either the geometric mean of TBP, GUSB, and YMHAZ, to beta-actin and to either 18S RNA for Sox9 or GAPDH for IGF-1. As can be seen in Fig. 4, when normalizing to the Geom mean, the expression of Sox9 is approximately 8-fold up-regulated after three weeks of chondrogenic induction when compared to levels at passage 1. Normalizing to 18S rRNA or beta-actin leads to a 2-fold under- or overestimation of Sox9 transcript levels, respectively. More dramatic results were obtained in the analysis of IGF-1 induction by hypoxia. As seen in Figure 4, when the cells are exposed to mildly hypoxic conditions for a week, the levels of IGF-1 message increase almost 2-fold. However, when normalizing to expression levels of GAPDH, which is strongly regulated by hypoxia, it erroneously appears that IGF-1 is down-regulated by hypoxic treatment. Conversely, when normalizing to beta-actin, an overestimation of IGF-1 induction occurs.

## Discussion

The recent decade has seen an almost explosive surge in the publications of research on adipose-derived stem cells. Understanding the differentiation of these cells will come about in a collaborative effort between a multitude of laboratories using different approaches. For a research group to build on the results of others, there must exist reliable data on gene expression patterns during cell expansion and differentiation. For the determination of even minute changes in mRNA expression levels, real-time RT-PCR technology has proven a powerful tool. However, as we have shown in this paper, to reliably determine small changes in expression levels in a quantitative manner, great care must be taken to select a number of reference genes for normalization purposes, thus limiting the effect of sample to sample variations.

In this study, we have identified a number of reference genes, which are suitable for gene expression studies in adipose-derived stem cells exposed to a variety of growth conditions. In the study by Gorzelniak et al (2001), on selection of reference genes to study gene expression in preadipocytes and mature adipocytes exposed to different hormones, the authors demonstrated that for that particular experimental set-up, GAPDH and transferrin were the most suitable gene for normalization [13]. In our study, we have shown that the expression of GAPDH is downregulated during chondrogenesis and upregulated in hypoxic conditions, yet is in the other conditions relatively stable, confirming the previous data. Other research groups have demonstrated the suitability of using 18S rRNA as reference genes in research on adipose tissue samples [14]. Complementing those results, we have in this paper shown that during the culture and passaging of primary ASC cultures, the levels of 18S rRNA increase at least two-fold from the initial values. In addition, we demonstrated the effect of choosing an inappropriate housekeeping genes, by normalizing the expression levels of Sox9 and IGF-1 to different housekeeping genes. The choice of reference gene had dramatic effects on the quantitation of changes in gene expression. Notably, using GAPDH for normalization led to the erroneous conclusion that the expression of IGF-1 is down-regulated during hypoxia. Our results further underscore the need for any researcher to carefully select reference genes prior to gene expression studies. In particular, if no studies have previously been done that compare gene expression levels for that particular experimental set-up, a small number of suitable reference genes should be identified. In our study we have chosen to select the reference genes by the GeNorm algorithm. A recent study comparing three different algorithms for detecting least variable genes, demonstrated only minor differences between the ranking of genes using these different methods [19]. Based on our results and those of other groups, we recommend testing a small subset of genes encompassing PPIA, YMHAZ, TBP, and GUSB, and selecting the two to three least variable.

## Conclusion

For studies on gene expression during mesenchymal stem cell growth and differentiation, adipose-derived stem cells are increasingly being used. For detection of small variations in gene expression levels in these cells, real-time quantitative RT-PCR is the method of choice. However, for the accurate determination of differences in transcripts between samples, a valid normalization strategy must be employed. In this study, we have demonstrated that several of commonly used reference genes including 18S rRNA and beta-actin are unsuitable for normalization during growth and differentiation of primary cultures of adipose-derived stem cells. We have identified four suitable reference genes, and recommend normalizing expression values of genes of interest to the geometric mean of at least two of these. Using this subset of reference genes allows for a reliable quantitation of even small gene expression changes in the cells.

## Methods

### Isolation and growth of adipose tissue derived stem cells

Subcutaneous adipose tissue was obtained from patients undergoing elective surgical procedures at Grymer Private Hospital, Aarhus, Denmark. The protocols were approved by the regional Committee on Biomedical Research Ethics in Northern Jutland. Cells were isolated from the adipose tissue essentially using methods previously described [20]. Briefly, harvested tissue was washed repeatedly with phosphate-buffered saline (PBS) and then enzymatically dissociated with 65 units/mL crude collagenase (Wako, Richmond, VA) in PBS supplemented with 20 mg/mL bovine serum albumin (BSA) at 37°C for 60 min. After incubation, the stromal vascular fraction of cells was pelleted by centrifugation at 400 g for 10 min. To remove contaminating erythrocytes, the pellet was resuspended in distilled water for 10 s after which the salt balance and pH were restored to physiological levels. The suspension was filtered through a 100-μm nylon mesh to remove cellular debris, where after the cells were collected by centrifugation, filtered through a 70-μm nylon mesh, and incubated overnight at 37°C/5% CO2 in control medium (DMEM/F12, 10% FBS, 100 u/ml penicillin, 0.1 mg/ml streptomycin, and 0.05 mg/ml gentamicin). Following incubation for 24 hours, all medium was changed to remove residual nonadherent mononuclear cells. The cells were maintained at 37°C/5% CO2 with medium changes twice a week. When cells were confluent, they were passaged at a ratio of 1:3. All subsequent studies on cell growth and differentiation were carried out twice and in duplicate.

For studies of changes in gene expression during passaging of the cells, RNA was isolated from cells at passages 0, 1, 2, 3, 4 and 5. Briefly, during the subculturing procedure, aliquots of the cells were seeded into 6-well plates (Corning, Costar, Acton, MA) at a concentration of 2 × 10^5 ^pr well in two mL of media and cultured for two days, after which RNA was harvested.

### Hypoxic treatment

Prior to hypoxic treatment, the ASCs (passage 1) were seeded in 6-wall plates at a concentration of 2 × 10^5 ^cells pr well in two mL of media. One day after seeding, the cells were transferred to a hypoxic workstation (Xvivo, BioSpherix, Redfield, NY), and incubated in an atmosphere with 1% oxygen and 5% CO_2 _balanced with nitrogen for up to two weeks.

### Chondrogenic differentiation

The ASCs (passage 1) were seeded in 6-well plates at a concentration of 2 × 10^5 ^cells per well in two mL of media and cultured in control medium until confluence. To induce chondrogenesis, the medium was switched to chondrogenic induction medium, consisting of high-glucose (4.5 g/l) Dulbecco's modified Eagle's medium (Invitrogen, Taastrup, Denmark) supplemented with 10 ng/ml transforming growth factor β3 (TGFβ3) (R&D Systems, Oxon, UK), 10^-7 ^M dexamethazone (Sigma-Aldrich, Broendby, Denmark), 50 μg/ml L-ascorbic acid 2-phosphate (Sigma-Aldrich), 40 μg/ml L-proline (Sigma-Aldrich), 100 μg/ml sodium pyruvate, 1× ITS^+ ^Premix (BD Bioscience, Brøndby, Denmark). After three weeks, RNA was harvested from duplicate wells and other wells were used for histochemical staining.

### Osteogenic differentiation

ASCs were seeded in 6-well plates at a concentration of 2 × 10^5 ^pr well in two mL of control medium. After 24 hours, osteogenic differentiation was induced by culturing ASCs in osteogenic medium (control medium supplemented with 0.1 μM dexamethasone, 50 μM L-ascorbic acid 2-phosphate, 0.5 μM calcitriol, and 10 mM glycerol 2-phosphate, all from Sigma-Aldrich). After three weeks, RNA was harvested from duplicate wells and other wells were used for histochemical staining.

### Adipogenic differentiation

ASCs in passage 1 were seeded in 6-well plates at a concentration of 2 × 10^5 ^pr well in two mL of control medium. After 48 hours, adipogenesis was induced by culturing ASCs in adipogenic medium (control medium supplemented with 0.45 mM isobutyl methylxanthine (Sigma-Aldrich), 170 nM insulin (Invitrogen), 0.2 mM indomethacin (Sigma-Aldrich). After two weeks, histochemical stainings were carried out.

### Histochemical stainings

Chondrogenesis was confirmed using the stain Alcian blue (1 g/L in 0.1 M HCl) (Sigma-Aldrich) for 30 min at room temperature. Before staining, the chondrogenic cultures were fixed in 4% formaldehyde for 15 min and washed with several changes of PBS. The calcium deposits in cells undergoing osteogenesis were stained with Alizarin red S (Sigma-Aldrich). The cells were rinsed in PBS, fixed in ice-cold 70% ethanol and incubated with Alizarin red solution (2 g/100 mL in distilled water, pH 4.2) for 15 min, after which the wells were rinsed repeatedly with water. Adipogenesis was assessed by staining the accumulation of intracellular triglycerides with Oil red O essentially as described previously [21]. Briefly, the cells were fixed in 4% formaldehyde for 1 hour, washed repeatedly with PBS, after with they were incubated the Oil red O working solution for 15 min, and then washed with water. The Oil red O working solution was freshly prepared from 6 mL of 0.5% (w/v) Oil red O in isopropanol mixed with 4 mL of water.

### RNA isolation and cDNA synthesis

Total RNA was isolated using the Aurum Total RNA mini kit (Bio-Rad, Copenhagen, Denmark) according to manufacturers instructions. Briefly, the cells were lyzed in 250 μL of lysis buffer, after which the RNA was immobilized on a silica membrane, washed, DNAse I treated and eluted in 80 μl RNase-free water by centrifugation. The purity of the RNA was determined by measuring the absorbance A_260_/A_280 _in a Nanodrop spectrophotometer. The quality of the RNA was assessed using lab-on-a-chip electrophoresis technology (Agilent 2100 Bioanalyzer, Naerum, Denmark). On the basis of 1 μg of RNA, cDNA was synthesized using the iScript cDNA synthesis kit (Biorad) according the manufacturer's instructions.

### Quantitative real-time PCR

The reference genes, tubulin, beta-polypeptide (TUBB), glyceraldehyde-3-phosphate dehydrogenase (GAPDH), beta-actin (ACTB), beta-2-microtubulin (B2M), ubuiquitin C (UBC), 60S acidic ribosomal protein P0 (RPLP), cyclophilin A (PPIA), tyrosine 3/tryptophan 5-monooxygenase activation protein (YMHAZ), TATAA-box binding protein (TBP), beta-glucuronidase (GUSB), hypoxanthine-guanine phosphoribosyltransferase (HPRT1) as well as rRNA 18S (RRN18S), were all included in the Human Endogenous Control Panel from the TATAA Biocenter (DNA-technology, Aarhus, Denmark). For the analysis of the differentiation of the stem cells, we analyzed the expression of the chondrocyte related transcription factor Sox9 and the insulin-like growth factor 1 (IGF-1). Primers for these genes were designed in-house using the PrimerSelect program of the Lasergene software package (DNA-STAR, Madison, WI) and manufactured by DNA-technology. The forward and reverse primers for Sox9 were 5'-CAC ACA GCT CAC TCG ACC TGG-3' and 5'-TTC GGT TAT TTT TAG GAT CAT CTC G-3', respectively, and the forward and reverse primers for IGF-1 were 5'-GAT GGG GTC TCG CAC TGT CCC-3' and 5'-GAG CCG AGA TCA TGC CAC TG-3'.

Quantitative PCR was performed on a My-Cycler real-time PCR system (Bio-Rad, Hercules, CA). The reactions were carried out in duplicates using the SYBR Green PCR supermix (Bio-Rad), using the manufacturer's instructions. The final reaction volume was 25 μL, and 5 pmol of primers and 0.25 μL of cDNA were used in each reaction. Each assay also included a blank. The PCR protocol consisted of an initial step at 95°C (3 min) followed by 40 cycles of 15 seconds at 95°C for DNA denaturation and 30 seconds of annealing and elongation. The annealing/elongation step was carried out at 60°C. To confirm product specificity, a melting curve analysis was performed after each amplification. The relative expression of each gene in the samples was calculated on the basis of a four-fold serially diluted standard curve derived from a pool of all the cDNA samples.

### GeNorm Analysis

To find the most stable housekeeping genes, we used the geNorm software package [4], which uses an algorithm to calculate the relative variation (M) of a given housekeeping genes to the remaining control genes. During this process, the most variable gene is excluded and the relative variation of the remaining genes is calculated. Secondly, also using the geNorm software, we determined how many reference genes should minimally be used for normalization purposes. This determination is based on the stepwise inclusion of housekeeping genes in the calculation of the geometric mean of the genes. For a more detailed description of the geNorm software, please refer to the excellent article by Vandesompele et al, 2002[4].

### Statistical analysis

For the comparison of the data normalized to the geometric mean of TBP, GUSB, and YMHAZ, to beta-actin and to either 18S RNA for Sox9 or GAPDH for IGF-1, first the data was confirmed to have a normal distribution, after which the data sets were compared using Student's t-test. Differences were deemed statistically significant with p < 0.05. All calculations were done with the aid of the exact tests package of SPSS 11.0 software (SPSS, Chicago, ).

## Authors' contributions

TF: conception and design, collection and assembly of data, data analysis and interpretation, manuscript writing. PL: collection and assembly of data regarding the ostogenic induction of the stem cells, manuscript writing. LP: collection and assembly of data regarding chondrogenesis, manuscript writing. JGR: manuscript writing MD: manuscript writing. VZ: conception and design, manuscript writing, final approval of manuscript. All authors read and approved the final manuscript
